# The Ecology of Bacterial Genes and the Survival of the New

**DOI:** 10.1155/2012/394026

**Published:** 2012-07-31

**Authors:** M. Pilar Francino

**Affiliations:** ^1^Unitat Mixta d'Investigació en Genòmica i Salut, Centre Superior d'Investigació en Salut Pública i Institut Cavanilles de Biodiversitat i Biologia Evolutiva, 46020 València, Spain; ^2^School of Natural Sciences, University of California, Merced, CA 95343, USA

## Abstract

Much of the observed variation among closely related bacterial genomes is attributable to gains and losses of genes that are acquired horizontally as well as to gene duplications and larger amplifications. The genomic flexibility that results from these mechanisms certainly contributes to the ability of bacteria to survive and adapt in varying environmental challenges. However, the duplicability and transferability of individual genes imply that natural selection should operate, not only at the organismal level, but also at the level of the gene. Genes can be considered semiautonomous entities that possess specific functional niches and evolutionary dynamics. The evolution of bacterial genes should respond both to selective pressures that favor competition, mostly among orthologs or paralogs that may occupy the same functional niches, and cooperation, with the majority of other genes coexisting in a given genome. The relative importance of either type of selection is likely to vary among different types of genes, based on the functional niches they cover and on the tightness of their association with specific organismal lineages. The frequent availability of new functional niches caused by environmental changes and biotic evolution should enable the constant diversification of gene families and the survival of new lineages of genes.

## 1. Introduction

Genomic science has brought about the possibility of disclosing the genetic underpinnings of entire organisms, and, for microbes, even of complex populations and communities. In doing so, it has unearthed a good number of surprising facts regarding the structure, organization, and variability of genomes, with strong evolutionary implications. In bacteria, the relatively small size of genomes has warranted the obtention of entire genomic sequences for a vast number of organisms, including numerous sets of sequences of closely related taxa. As genome sequences of closely related bacteria have accumulated, our view of bacterial genomes has radically changed. 

Genome comparisons have demonstrated that little 16S rRNA sequence divergence can be accompanied by large differences in total gene repertoire [1–7], and that even populations of a single 16S rRNA species can be made up of vast numbers of genomic varieties [8]. Much of the observed variation among closely related bacterial genomes has been attributed to gains and losses of genes that are acquired horizontally, often by way of mobile genetic elements (MGEs) as well as to a variety of genomic rearrangements that include gene duplications and large gene amplifications [1, 2, 4–6, 9]. This wide extent of genomic variability speaks to flexible and dynamic genomes and undoubtedly contributes to the amazing ability of bacteria to survive and adapt to varying environmental challenges. On the other hand, from a gene-centric perspective, the duplicability and transferability of individual genes imply that natural selection should operate, not only at the organismal level, but also at the level of the gene. It has long been recognized that MGEs can evolve selfishly [10, 11]. However, because of HGT, the distinction between selfish, mobile DNA and the sedentary host genome is blurry and it can be argued that all genes are semiautonomous entities capable of responding to natural selection at different levels: selfishly, because they can “reproduce” by duplication and “migrate” by horizontal gene transfer (HGT), and cooperatively, because their long-term survival depends on their ability to be replicated as parts of integrated genomes [12].

## 2. Genes as Ecological Semiautonomous Entities: The Gene's Functional Niche

Given the ubiquity of MGEs and the numerous HGT mechanisms in operation in the prokaryotic world, a given gene may be transported to any number of organisms in a variety of environments. However, not all of these may represent suitable venues for the incoming gene to persist. To be stably maintained in the long term, a gene would need to encode a function that conferred a valuable adaptation to the organism. An incoming gene not providing a novel capability to the recipient would not be selected for, not spread in the recipient population and eventually be obliterated by the high substitution and deletion rates operating in prokaryotic genomes [13–15]. So, a gene can be considered to have a potential range of genomes in which it could survive, by means of providing a selectable function. The functional need covered by the gene can be considered its very own niche, the defined functional space that would allow it to survive in the range of genomes where such a need exists.

 Following this analogy, genes will have, as do organismal species, fundamental and realized ecological niches. The fundamental niche of a gene can be defined as the entire range of genomes where such a gene might survive, in case it were capable of reaching them and they did not contain a similar gene realizing the same function. The realized niche will encompass the actual range of genomes where the gene has been established. In many cases, the realized niche of a gene will likely be substantially narrower than its fundamental niche. Besides physical limitations to gene dispersal, this could result from the fact that many populations of potential hosts may contain a better-adapted gene occupying or competing for the same functional niche, be it an ancestral gene or a different gene acquired by HGT. The following paragraphs explore the different types of gene niches and the likelihood that they are realized by horizontally acquired genes. 

### 2.1. Housekeeping Ancestral Functions

Gene functions that are essential for life are usually covered by ancestral genes that have been associated with their host genomes throughout evolutionary history, being passed on through generations via vertical inheritance [16, 17]. Since they are already represented in every living organism, there is no empty niche available into which these genes might expand, and therefore their realized niche is identical to their fundamental niche. Moreover, the long-term permanence of these genes within their specific genomes should have enabled a high degree of coadaptation with other stable members of the genomic community, favoring the evolution of a well-integrated gene core. Therefore, the niches represented by these functions are likely to be stably occupied by finely adapted genes that should be almost impossible to displace by incoming, horizontally transferred genes evolved in different genomic backgrounds. So, given that empty niches are not available and that displacement of orthologs in different genomes should be unlikely, horizontal transfer events should rarely be successful for these genes.

### 2.2. Ecologically Restricted Essential Functions

Functions that are required for survival or that substantially improve fitness only for specific habitats and/or lifestyles represent niches that may be available to horizontally transferred genes under certain circumstances. Such functions are often first acquired by HGT [18] so that the level of coadaptation between the gene that occupies the functional niche and its genomic companions will depend on the time elapsed since the HGT event occurred and the organism in question adopted its current lifestyle. Genes that were recently acquired by an organism and enabled it to occupy a new niche may have become indispensable for the organism's survival, but may not be better suited to their role than genes sharing the same fundamental niche present in the genomes of other species. In such cases, competing incoming genes may have a selective advantage and be able to replace the original gene. In addition, nonhousekeeping functional needs, even when essential, may be more sensitive to varying environmental conditions and be altered as organisms adapt to a new environment or radiate into a range of similar lifestyles. These circumstances should also provide opportunities for displacement of the resident genes by newcomers transferred horizontally. 

### 2.3. Nonessential Accessory Functions

A large fraction of the genes unveiled by sequencing projects is present across only some of the genomes of a given bacterial species. This gene fraction has been termed the dispensable or accessory genome and can amount to a large proportion of the total gene repertoire detected across the species, the pangenome [3]. It includes numerous functions that are not generally required for survival, but that may ameliorate adaptation to transient conditions or patchy environments. Shifting environmental conditions should often generate vacant functional niches, transiently made available to a variety of existing genes capable of fulfilling the required role after horizontal transfer. Many factors might come into play in determining which of the possibly numerous genes sharing the available fundamental niche will eventually occupy it. Such factors should include the physical proximity and phylogenetic relatedness among potential donor and recipient organisms, which increases the likelihood of a successful gene transfer [19, 20] as well as the likelihood that the gene might be associated with MGEs capable of reaching the potential recipient. If different genes were transferred, competition among them should ensue and the one better adapted to the available functional niche should spread more widely into the recipient population. 

### 2.4. Selfish, Gene-Centric Functions

Some genes may not require a preexisting niche provided by a functional need at the organismal level. This is probably best exemplified by systems that behave as addiction modules, or poison/antidote systems, which commonly spread via plasmid-mediated HGT [21]. Such systems consist of two genes acting as a toxin and an antitoxin for the cell that carries them. The toxin kills cells if expressed above a certain level, and the antitoxin inactivates the toxin and/or regulates its expression, thereby preventing cell killing. The toxin is more stable and long-lasting than the antitoxin so that constant (over)production of antitoxin is required for cell survival. Thus, toxin/antitoxin systems can be said to carve their own niche into the genome, as their permanence is ensured independently, and possibly in detriment, of organismal-level requirements [22, 23]. Although not mobile, these two-gene systems can be considered selfish in the sense that their primary function is self-preservation, similar to insertion sequences and other transposable elements [10, 11].

## 3. Creation of Functional Niches and Survival of the New

Most importantly, novel functional niches are likely to appear constantly, as environments change and species evolve, enabling the evolution of novel genes. Although radically new biochemistries and structural functions may be required more rarely, specialization of preexisting functions to meet modified organismal needs should often be advantageous. In addition, any existing functional niche could likely be subdivided into multiple narrower niches, which could be occupied by genes with higher degrees of functional specialization. Numerous cases of specialization of different homologs into related but distinct functional niches have been documented, including the evolution of affinity for different substrates or cofactors [24–28], of different enzymatic kinetic profiles [29, 30] and of different interaction patterns with other proteins [31]. Functional niches for homologous genes with different specializations can be available within single organisms, when their lifestyle encompasses variable environments [32], different developmental stages [33] or dynamic interactions with hosts [31]. In fact, antagonistic biotic interactions, such as those between pathogenic bacteria and their hosts or those between bacteria and their phages, can lead to arms races that permanently favor diversity [34]. Such processes should constantly generate new niches for bacterial genes, differing from existing ones by the novel constraints imposed by changes in the interacting organism. Clearly, similar but distinct gene niches can also be generated when different organisms specialize into different subdivisions of an organismal niche, as occurs during adaptive radiations. A beautiful example of concordant gene and organismal radiations has recently been described within soil archaeal ammonia oxidizers, where clades of *amo*A, a key functional gene of ammonia oxidation, dominate specific ranges of soil pH [35]. 

 Functional niches likely to be substantially different from those covered by existing genes are also being created today by the plethora of human-made compounds released into the environment. Enzymatic pathways capable of degrading some of these compounds have readily evolved, including pathways for the degradation of the pesticides atrazine [36], pentachlorophenol [37, 38] and 1-3-dichloropropene [39], and of chloronitrobenzenes and dinitrotoluenes used in the production of industrial chemicals and pharmaceuticals [40, 41]. Most of these chemicals are highly toxic and mutagenic, which has likely represented a strong selective pressure for the rapid evolution of degradation pathways. However, many human-made compounds are highly recalcitrant to degradation and have only been present in the environment for a few decades so that evolution has had limited time to produce genes and enzymes to fill the novel niches they provide. For example, no naturally occurring microbes are known to completely mineralize the dielectric fluid PCB or the insecticide paraoxon although they can be partially detoxified. The products of extant reactions of detoxification or incomplete degradation of anthropogenic chemicals, such as the partially dechlorinated compounds resulting from reductive dehalogenation of PCB [42], can accumulate in the environment and provide suites of novel functional niches. Some anthropogenic compounds cannot be degraded by any single organism, but their degradation can be accomplished by enzymes from different microbes working together, as occurs with the explosive trinitrotoluene (TNT). In this case, none of the participant microbes carries out enough of the involved reactions to reap a metabolic benefit from TNT degradation [43]. Therefore, there still remains a niche, or niches, for the evolution of integrated functional pathways within single microbes that can utilize TNT as a novel source of carbon, nitrogen, or phosphorous. 

A special case of human-generated selective pressure for the evolution of novel bacterial gene functions is provided by the widespread use of antibiotics. Although some antibiotic resistances may have preceded the use of antibiotics by humans, serving to defend bacteria from chemical warfare from other microbes, it is clear that the large variety of resistance genes existing today attests to a rampant diversification. Novel resistance genes evolve and spread rapidly, with the typical timeframe for worldwide dissemination of a newly emerged gene being under three years from the initial deployment of the antibiotic [44, 45]. 

 How are the partition of functional gene niches and the occupation of novel ones achieved, often in record times? The capacity of proteins to alter their substrate ranges has been well documented during experimental evolution [46, 47]. Enzymes subject to directed laboratory selection under high rates of mutation and recombination rapidly increase their level of activity on new substrates by several orders of magnitude over the wild type. On the other hand, computational analyses of molecular dynamics indicate that the substrate range of an enzyme can just as easily be narrowed down. For instance, the alkaline phosphatase of *Escherichia coli* acts on both phosphomonoesters and phosphodiesters through a single-reaction mechanism, but simulation studies indicate that specialization for hydrolysis of mono- or di-esters, seen in other members of the alkaline phosphatase superfamily, depends on mutations that alter the nature or positioning of a single amino-acid residue [48]. Thus, niche partitioning may often result from the small alteration of existing gene functions, which could come about through a small number of mutational events. The occupation of new niches can also be enabled by small modifications that alter enzyme specificity. For instance, in *Pseudomonas diminuta*, a phosphotriesterase enzyme specialized in the utilization of pesticides is thought to have evolved recently from an amidohydrolase endowed with several promiscuous activities, and experiments with a homologous amidohydrolase have shown that the substitution of a single amino-acid can radically change the specificity of the enzyme [29]. The evolution of novel functions that are more distinct from preexisting ones may necessitate a wider arsenal of genetic alterations. As an example, the newly evolved enzymes that degrade chlorinated aromatics in the environment bear in their sequences the hallmarks of complex genetic rearrangements, including recombinational events mediated by transposable elements [49]. 

 The accumulation of mutations that generate novel functions usually occurs during divergence between homologous genes. The process of divergence can be initiated in paralogy, after gene duplication within a given genome, or in orthology, following organismal speciation. The following section explores the potential differences between these two modes of evolutionary divergence and their consequences for the evolution of new functions as well as the role of processes that reassort the evolutionary novelties generated during divergence.

## 4. Genes as Phylogenetic Semiautonomous Entities: Gene Lineages

### 4.1. Orthologous Gene Divergence

After organismal speciation, newly created pairs of orthologous genes will start to diverge, at a rate that will depend, among other factors, on the degree of recombination between the two daughter species at that specific gene locus. In bacteria, the capacity of genetic exchange across species boundaries implies that orthologous genes may continue to recombine for a certain time after two incipient species have formed, and even among closely related species, at rates dependant on the amount of sequence divergence at that locus and on the potential fitness decrease caused by recombinant gene products [50–52]. If the species remain in contact, recombination between orthologous genes may continue as long as the accumulated level of sequence divergence does not pose an impediment for homologous recombination (HR) mechanisms [53–55]. If the organismal niches occupied by the two species do not pose different selective pressures on a given gene locus, most of the divergence that will accumulate between orthologs should be neutral, and functional divergence should not occur, barring the potential fixation of deleterious changes due to drift. If no functional divergence occurs, recombinant gene sequences are likely to be equally fit as the parental ones, at least until species-specific patterns of codon usage or other sequence-level adaptations begin to take place.

 In contrast, if the organismal niches of the daughter species represent different functional niches for a given gene, divergence should occur under positive selection on one or both orthologs. In this case, recombination events between orthologs may produce sequences that are less fit in their corresponding niches than the parental ones and should be selected against. This should facilitate further divergence and specialization of one or both orthologs for their specific functional niches. As a result, such orthologs may be considered different evolutionary entities, as they fill different functional niches and evolve as separate lineages not incurring genetic exchange. 

### 4.2. Paralogous Gene Divergence

The processes of gene duplication or amplification will generate gene copies that are identical to each other and often clustered within a genome [56, 57]. Although genetically unstable due to loss by HR, such sets of identical paralogs may be maintained in the genome if the increase in the amount of gene product is beneficial to the organism [57–62]. Although the fitness effects of most duplications, as those of other mutations, will likely be deleterious or neutral, there is ample evidence that gene amplification and increased production of the encoded protein can occasionally provide specific selective advantages. Adaptation by means of gene duplications and larger amplifications has been repeatedly documented in bacteria. Gene amplifications have been implicated in enhanced virulence in pathogens as well as in increased production or fixation of host-required nutrients in symbionts. Gene amplification has also been demonstrated to underlie instances of bacterial resistance to antibiotics and heavy metals [63], as well as experimental adaptation to growth at high temperature [64], and on limiting or unusual carbon sources [65, 66]. Such sets of paralogs that continue to occupy the same functional niche will evolve more or less cohesively, depending on the level of recombination among the different copies. Divergence among them may actually be deleterious so that change-of-function mutations would be selected against while gene conversion events that maintained the original sequence would be selected for. 

 However, it seems unlikely that two or more identical genes would be maintained in long-term evolution for the purpose of increasing the amount of gene product. To that effect, alternative, probably more efficient strategies would be available to an organism, such as increases in the expression of one gene copy through regulatory changes. “Clonal” gene amplifications are, therefore, likely to be transient in nature. Analyses of gene family sizes indicate that the amplifications with the largest numbers of gene copies are usually very recent [61, 67–72], and calculations of the age of appearance of gene duplicates within bacterial clades indicate that most of them are young [9], implying that, overall, most paralogs do indeed disappear rapidly. If higher gene dosage is the only pressure that maintains gene amplification, the high deletion rates operating in bacteria [13–15] should favor the rapid elimination of superfluous gene copies once regulatory variants producing higher amounts of product appear, or if selection for higher dosage wanes. In such cases, most of the gene clones can be expected to eventually disappear, unless they are rescued by adaptive mutations that allow them to occupy a novel functional niche. 

 If there is formation of new, separate niches, new functions can evolve and genic lineage diversification will occur. Selection for higher gene dosage may actually often respond, not to a requirement for higher levels of a gene product's extant function, but to a novel functional need that can be partially accomplished by the amplification of an existing gene. The evolution of the new function may start with the amplification of a gene having some level of preadaptation for that function. Gene amplification would provide the means to attain biologically significant levels of functionality, as the efficiency of the preadapted gene product for the new function would presumably be low [58, 61]. Amplification could be followed by positive selection to adapt the gene product to the novel requirements. A period of competition among the different evolving paralogs in the population might ensue, resulting in the preservation of the most effective variant and the likely pseudogenization and eventual loss of the rest [61]. As in the case of orthologs adapting to different niches, recombination among paralogs diverging in function should probably be selected against, facilitating their separation into independently evolving gene lineages. There is ample evidence that paralogous gene amplifications have resulted in diversified functions of high adaptive value, including expansions of metabolic and regulatory capabilities [28, 73, 74], sensory complexity [75], or antigenic variation [76]. 

### 4.3. Advantages of Paralogous Divergence for the Diversification of Gene Function

Both gene duplication and speciation are processes that enable cladogenesis, the generation from a common ancestor of new evolutionary entities that occupy different functional niches and evolve as separate lineages. In orthology, however, the new gene lineage created by the splitting-off of a new species may remain constrained by similar selective pressures if the functional need it served in the ancestor remains unchanged. In time, the new gene lineage will likely adapt to its specific genomic and environmental context, but significant functional divergence should occur only if the lifestyle of the novel species changes substantially and in a manner that alters the functional need served by the gene. In contrast, gene duplications and larger amplifications generate a number of replicas of the same gene within a single organism, so that each one of them may be free to engage in different evolutionary paths, including the retention of the original function in one or a subset of the gene copies. 

 In particular, the generation of new gene functions may be most facilitated in the context of large gene amplifications that are positively selected from their inception. As mentioned above, amplification of an existing gene with some level of preadaptation to the newly required function may be a first adaptive strategy when an organism is confronted with a new functional need. In this case, the duplicates would be maintained by natural selection, ensuring the permanence in the population of a large number of gene copies that could be the target of mutations with a potential ameliorating effect on the novel function. Moreover, the existence of multiple gene copies would enable the simultaneous exploration of different zones of the adaptive landscape, including fitness valleys that might allow them to transition into separate adaptive peaks, potentially leading to distinct functions. During this process, the existence of related gene sequences within the same genome, and likely in proximity to one another [62], would allow for recombination to occur, which, although potentially hindering the process of divergence, might also bring together beneficial mutations acquired in different paralogs or purge those that are detrimental. Finally, this mode of evolution might be reinforced by the sequential acquisition of beneficial mutations alternating with rounds of selected amplifications of the best-adapted paralogs at every step. In the long term, if new but related functional niches continue to appear due to changes in the environment or in the organism's lifestyle, families and superfamilies of paralogous genes may be generated in adaptive radiations analogous to those observed for species lineages [61]. 

### 4.4. Mixing It Up: Recombination Among Genes and HGT across Organismal Lineages

Once novel genes with distinct functional niches have evolved, in orthology or in paralogy, they may be available for filling those niches in other organisms through HGT. As evidenced through the sequencing of bacterial isolates and environmental metagenomes, the divergence processes just described have created gene families that encompass an enormous variety of related sequences. Functional characterization of these sequences lags way behind their discovery, but it is likely that many of them encode related but distinct protein functions. Consequently, when a novel functional niche opens within an organism, a large number of existing related genes may be able to fill it in similar, but probably not identical, manners. Genes with functions similar to the one required could be encoded within the organism's genome so that the niche could be filled by the processes of amplification and divergence developed above. Alternatively, the niche may also readily be filled via horizontal acquisition of a foreign gene that might already be well-suited to the novel need. Functional niches that originate as organisms undergo substantial lifestyle modifications should be more often filled by HGT, given the capacity of this process to bring in functions radically different from those already encoded by the organism. Accordingly, HGT has been repeatedly documented to be a prime contributor to the adaptation of bacteria to novel environments [4, 18, 77–82]. Similarly, the adoption of symbiotic or pathogenic lifestyles, as well as their diversification in terms of host range and tropism, is most often enabled by the acquisition of genes encoded within MGEs [78, 83–94]. Moreover, bacterial subpopulations that gain access to very different niches by HGT may readily become independent lineages, and thus HGT can be considered a motor of prokaryotic speciation and long-term diversification [18, 50, 77, 78, 95, 96]. Amazing examples of appearance of major lineages with specific biologies introduced by HGT include the emergence of bacterial methanotrophs via acquisition of archaeal genes [97] and that of cyanobacteria via the gain of a second photosystem allowing for oxygenic photosynthesis, possibly transferred from the Firmicutes [98].

 Clearly, the existence of HGT enables the dissociation of gene and organismal lineages. By reassorting genes across organisms, HGT may constitute an important driver of further gene lineage diversification. Horizontally transferred genes will be exposed to different ecological and genomic environments and their associated selective and mutational pressures, which should favor divergence from the genes in the donor population. Several studies have confirmed that recently acquired genes have an accelerated rate of evolution in comparison to that of ancestral genes in the same genome [2, 88, 99, 100]. This could respond to relaxed selective pressure in genes not conferring a significant advantage to the host, to neutral substitutions due to the host's mutational biases, or to positive selection for adjusting gene expression or protein function to the specific needs of the host. It has been documented that, in some cases, proteins may undergo significant shifts in function after HGT. For example, omptins are a family of outer membrane proteases that have spread horizontally through a large variety of Gram-negative bacteria infecting vertebrates and plants, and their functions have been substantially modified to adapt to these different lifestyles [101]. Genes that have been acquired by HGT have also been shown to undergo more duplication events than ancestral genes [102]. Gene duplication following transfer could compensate for suboptimal expression or activity of genes and proteins that are not adapted to their novel genomic and cellular backgrounds and could facilitate the appearance of better-adapted gene copies or the evolution of paralogs displaying more substantial functional shifts. So, although gene duplication and HGT independently contribute to fill in new functional niches, and may be best suited to do so under different circumstances, the two processes may sometimes be coupled in the course of adaptation. 

 Another factor likely to impact the evolution and diversification of horizontally transferred genes in a substantial manner is the fact that these genes may spend significant periods of time in association with MGEs. This association should expose them to mutational pressures different from those affecting chromosomal genes due to the specific replication modes employed by such elements. In fact, most bacteria contain large numbers of ORFans, that is, annotated genes that are restricted to a particular genome and that possess no known homologs in any other organisms, and the sequence characteristics of these genes, including short length, high AT content, and often phage-like dinucleotide frequencies, have been considered to be hallmarks of substantial periods of evolution within phage genomes [2].

Besides sorting out entire genes across organisms, the capacity of bacteria for genetic exchange can also diversify gene lineages through HR among related genes that have diverged under orthology or paralogy. Although normally strongly constrained by sequence differences, HR can operate on more divergent sequences when the SOS system is induced, or in mutants of the methyl-directed mismatch repair system (MMRS) [103]. When operating on closely related genes, HR will mostly serve to limit their divergence, but the occasional exchange between diverged genes that have reached different functionalities can create novel capacities not present in the parental sequences. This phenomenon has been documented in the generation of novel resistance phenotypes among antibiotic resistance genes and is thought to have been a major source of diversification for genes such as *bla*_CTX-M_, *ampC*, and *qac* [45]. HR also generates functional diversity in gene families, such as the histidine kinases involved in signal transduction, with members that contain variable combinations of sequence domains, by enabling domain-shuffling among paralogs [74, 104]. In addition, MGEs encode more than a hundred different enzymes capable of recombining DNA at short specific nucleotide sequences, without the long stretches of homology required for HR [105], that could potentially contribute to the creation of novel genes by recombination of sequences from different origins. However, unlike the case of eukaryotes, where rearrangements promoted by MGEs contribute significantly to the generation of novel chimeric genes [106], this process should be rare in bacteria, as the uninterrupted nature of bacterial coding sequences must severely limit the chances of a foreign sequence being integrated without disrupting gene function. Nonhomologous recombination, though, may be an important driver of the evolution of new genes within bacteriophages and may contribute to the generation of the phage-derived ORFans that abound in bacterial genomes [107]. 

## 5. Emergent Genomes within Organismal Lineages

Within the described context of genes as ecological and phylogenetic semiautonomous entities that possess their own functional niches and undergo their own historical processes, the genome appears as an emergent gene community, a moving picture of gene associations, some of which will endure, while others will be transient in nature. The genome not only provides the material framework in which the genes are embedded, but also the information required to regulate their interactions. The result is a community of interdependent genes that operate as an integrated whole. This picture is similar to that of ecological communities of organisms, which are capable of maintaining regulated dynamics and species interactions even if their exact species composition fluctuates through time and space [12].

### 5.1. Variable Levels of Association between Gene and Organismal Lineages

Clearly, the length and tightness of the association between gene and organismal lineages can vary greatly, depending on the type of functional niche occupied by the gene. Genes that perform housekeeping ancestral functions are essential for life and usually remain associated with the same organismal lineage through vertical inheritance. These essential genes will evolve under strong selective constraints that will simultaneously penalize gene loss, accelerated sequence evolution rates, horizontal transfer and, for highly interactive proteins, gene duplication, in order to maintain a stable, host-specialized, and coadapted genomic core. Such constraints signify that the phylogenetic history of the genes of the universally essential genomic core should mostly parallel that of the organismal lineages where they reside. From the point of view of reconstructing the deep phylogenetic history of organismal lineages, these genes should represent the best available markers. Representative genes of this evolutionary mode are those involved in transcription and translation, which encode highly interactive coadapted protein complexes and rarely undergo HGT [108–114]. 

Beyond the core of genes essential for life, many genes may be stably associated with specific organismal lineages, even though they may have been first acquired by HGT in an ancestral species. The numbers and types of genes that are conserved increase significantly within progressively shallower phylogenetic lineages [115]. For instance, whereas all prokaryotes almost certainly share less than 50 genes, over 100 genes are common to all bacteria [116], and 205 single-copy genes are present across all the *γ*-Proteobacteria, a large and ancient group that originated over 500 million years ago [117]. Within the *γ*-proteobacterial enteric family, the core genomes of the large and well-characterized species *E. coli* and *S. enterica* have been estimated at around 1000 and 2800 genes, respectively [118, 119]. Besides informational genes, these species-level cores mainly include genes involved in the biosynthesis of aminoacids, nucleotides, cofactors and proteins as well as in the metabolism of DNA, fatty acids, and phospholipids [120]. However, genes that are ubiquitous within restricted phylogenetic clades, such as species or genera, may not be stably associated with specific lineages within the clade, but rather may undergo frequent horizontal shuffling within these groups, depending on the population structure and level of ecological divergence of the different lineages, the level of functional niche specialization of the gene, and the capacity of the organisms to incorporate exogenous DNA. 

 Finally, a large fraction of genes present erratic patterns of presence across different organisms, including closely related strains, and phylogenetic relationships that are indicative of frequent HGT among organisms with varying degrees of relatedness. Some of these accessory genes are clearly associated with narrow but defined niches or lifestyles, most notably in the case of pathogens and symbionts, where genes acquired horizontally within specific strains can remain in their genomes for long periods of time. However, many other accessory genes encode functions that may ameliorate adaptation to transient conditions or patchy environments. As such, their long-term permanence in a given organismal lineage is not warranted. Phylogenetic analyses confirm that most horizontally acquired genes are eventually lost [121–123]. In fact, the high rate of horizontal gene acquisition that has been documented for many bacterial genomes must have been compensated by a similarly high rate of gene loss, otherwise genomes would continuously increase in size. Therefore, the makeup of the accessory component of genomes must continuously fluctuate through time. Moreover, analyses of genomic diversity at the population level indicate that the presence of accessory genes can be variable even among cooccurring cells within local populations [4]. This local population-level variability suggests that these accessory genes are not stably associated with subspecific lineages adapted to particular but defined environmental conditions, that is, ecotypes [124], but rather that their presence in a given genome may respond to transient selective pressures in a variable environment. Extensive temporal and geographic sampling of natural microbial communities at variable scales may be able to reveal whether specific accessory genes are stably or preferentially associated with certain genomic variants, or whether their distribution indicates a more nomadic existence with frequent transfers among variants and/or across different organismal lineages [12]. 

 For genes that only maintain transient associations with specific genomes, long-term survival may be ensured by HGT across a variety of organisms that only occasionally require the function provided by the gene. The likelihood that gene lineages might survive by this strategy should be linked to (1) the fitness increase provided by the gene, (2) the pattern and scale of environmental variability, and (3) the transmissibility of the gene within and among organismal lineages. For instance, accessory genes that provide strong fitness advantages under transient conditions may temporarily reach high frequencies within local populations or communities, and their long-term maintenance may be ensured by their capacity to disperse to other microbial communities during such periods of high abundance. This may be the case of accessory genes that confer resistance to broad range antibiotics or other strong selective agents that affect a variety of species but have a patchy distribution. On the other extreme, accessory genes for which presence is neutral or even detrimental relative to organismal fitness may be able to survive via high levels of transmissibility. This scenario may be approximated by addiction modules, such as toxin-antitoxin systems, which have extensive horizontal mobility due to their associations with plasmids, phages, transposons, or integrons, whilemost likely presenting scarce benefits to organismal fitness [12, 22, 23], although potential roles for these systems in bacterial stress adaptation have been proposed [125, 126].

### 5.2. Enabling Gene Cooperation in Emergent Genomes

Whatever their origin, the different genes that coexist within a genome at a given time must operate within the larger context of this higher level of organization. The genomic framework in which the genes are embedded must encode the information required to orchestrate their function in order to face the demands of the environment. In addition, various structural organization constraints will affect the physical distribution of genes within the genome.

 Incoming genes should not be able to integrate at any genomic location. Clearly, gene insertions within coding regions and other functional sequences will often be deleterious, but more subtle aspects of genome organization also need to be considered, as genes are not randomly distributed within the genome, but are rather organized at several different levels. Many genes are part of operons, and the disruption of operon structure may negatively impact the coordinated expression of the constituent genes. Larger organizational domains are also present in bacterial chromosomes, associated with structural constraints imposed by the processes of DNA replication and segregation at cell division, the disruption of which strongly affects fitness [127–130]. Also, patterns of DNA supercoiling along the chromosome have been implicated in coordinating the expression levels of contiguous genes in a manner that varies along the bacterial growth cycle [131]. Therefore, the wide fluctuations in gene composition that characterize bacterial genomes need to preserve these fundamental structural properties. This may be accomplished in part by limiting changes to specific genomic regions. The observed variability among the genomes of closely related organisms is indeed often confined to a few genomic locations, while the overall genomic framework is tightly conserved [120]. In addition to the likely role of natural selection, sequences and/or genomic architectures that serve as hotspots for recombination can contribute to the generation of such a pattern. For instance, many genomic islands and phages integrate preferentially next to tRNA genes [132], and these sites often concentrate a substantial fraction of a genome's recently acquired genes.

 Bacterial genomes are also well integrated in terms of coadaptation among their different components, such as the molecules that participate in complex multimeric enzymes or structures. Another amazing example of coadaptation is that between the complement of tRNAs in a genome and the codon usage bias of its coding sequences. In many bacteria, the most highly expressed genes utilize restricted sets of codons that display optimal interactions with the most abundant tRNA species for a given aminoacid present in the genome, allowing for fast and accurate translation of their mRNA [133–137]. Genes from exogenous origin will display codon usages that are distinct from those of the host genome, especially if they are incoming from a genome of different GC content or if they have spent substantial amounts of time associated to phages, which have very elevated AT frequencies. Expression of foreign genes with a codon usage that is not matched to the recipient cell will then be compromised [138]. Accordingly, it has been shown that most recently acquired genes have a codon usage similar to that of the recipient genome at the moment of introgression, indicating that codon usage compatibility is likely to increase the fixation probability of transferred genes [139]. Nevertheless, experimental evidence shows that genes with poorly matched codon usages can be retained if they confer a strong selective advantage, and that their expression level can be rapidly adjusted by regulatory changes [138]. Moreover, the mutational and selective processes particular to the host genome should in time modify their codon usage towards patterns typical of ancestral genes [2, 140, 141]. 

 Most importantly, the expression of the assortment of genes present in the genome needs to be regulated and coordinated for meaningful biological function. Exogenous genes may arrive within the genome accompanied by cognate regulatory sequences and regulator genes. Examination of the evolutionary histories of transcription factors in *E. coli* indicates that many specific regulators were horizontally acquired along with the adjacent genes that they regulate, whereas global regulators are encoded by genes that evolved vertically within the *γ*-Proteobacteria. Also, horizontally transferred genes are often regulated by multiple regulators, with most of this complex regulation probably evolving after transfer [142], speaking to the likely existence of strong selective pressures to fine-tune their gene expression and integrate it within the context of global regulatory networks. As an example, the expression of virulence genes of the *Salmonella* SPI-1 and SPI-2 pathogenicity islands is controlled by a complex regulatory cascade involving several global regulatory systems as well as specific regulators [143, 144]. The integration of transferred genes into the host's regulatory network appears to occur gradually over evolutionary time, as genes resulting from increasingly ancient transfer events show increasing numbers of transcriptional regulators as well as improved coregulation with interacting proteins. In addition to the recruitment of existing transcription factors, increased integration is accomplished by sequence evolution of the cisregulatory regions and changes in the codon usage of the transferred genes [145].

 The topology of the networks of gene and protein interactions may facilitate or hinder the incorporation or loss of accessory genes. Comparative analyses have shown that gene networks often contain a core of ancestral genes involved in large numbers of interactions (hubs) that are highly conserved across species, while genes that are progressively acquired during evolution encode less connected and less central proteins. Therefore, regulatory [146], metabolic [147, 148] and protein interaction networks [146, 149] appear to grow by acquiring genes in the periphery. This network topology and mode of growth clearly enable a flux of accessory genes onto a core genomic framework, allowing for niche exploration and adaptation to changing environments. Another common property of gene networks that facilitates the exchange of accessory components is modularity. Bacterial regulatory and metabolic networks are often organized in well-defined modules, sets of genes or proteins that are strongly interconnected and with a function that is separable from those of other modules. This property is believed to be one of the main contributors to the robustness and evolvability of biological networks [150]. Simulation analyses have shown that modularity allows for specialization in gene activity because it decreases interference between different groups of genes and facilitates cooption, the utilization of existing gene activity to build new functional patterns [151].

 Clearly, genomes are shaped by structural and organizational properties that ensure their existence as coherent levels of organization in the face of the malleability conferred by gene duplicability and horizontal transfer. Such properties can be considered emergent properties [152] of the genome because they ensue from the relationships among its different components (sequences with coding, regulatory, or structural functions) and shape its global interaction with the environment. Emergent genome properties will be acted upon by natural selection at the organismal level, which will eliminate unfit combinations of genes or interactions. Moreover, organismal-level selection should favor the appearance, maintenance, and refinement of emergent genome properties, such as genomic architectures and gene network topologies, that enable the organized gain, loss, and reshaping of functional capacities to facilitate organismal adaptation, specially under conditions of frequent environmental change. 

## 6. Concluding Remarks

Beyond gene and organismal selection, the exchange of genes among individuals from different species generates genetic relatedness between them and further diversifies the levels of organization at which natural selection may act. In particular, by increasing relatedness at transferred loci, HGT could favor the evolution of cooperation among gene-exchanging individuals [153–156], although differences in relatedness between mobile loci and the rest of the genome raise the possibility of conflict regarding what type of interaction with a given neighbour might be most advantageous. More generally, genes that undergo frequent transfers among organismal lineages represent a supraspecific gene pool that can increase the fitness of individual organisms belonging to different species. In a given microbial community, organisms from many different species may have access to the same supraspecific gene pool, which will be composed of transferable genes present in the genomes of the community's microbes and MGEs, or brought in by immigrants from other communities. For instance, metagenomic analyses of the human gut microbiome have evidenced that identical antibiotic resistance genes can be shared by bacteria belonging to different bacterial phyla within a single individual [157]. In addition, the MGEs present in the community will largely shape the type, rate, and directionality of the HGT processes that will distribute the gene pool. Under certain conditions, such as those involving frequent environmental fluctuations, the survival of entire communities may depend on their metagenomic pool of transferable, accessory genes, and on the type and rate of HGT processes that may distribute it among organisms facing similar selective pressures [12]. Therefore, the supraspecific pool of accessory genes and the community's capacity for HGT should be considered emergent community properties that may enable the operation of natural selection at the community level. 

 So, in the bacterial world, the capacity of genes to duplicate and to transfer among organismal lineages enables natural selection to proceed at several different levels, including that of the gene, the organism and, possibly, the community. Selective pressures operating at the gene and organism levels result in the diversification of gene lineages to track new functional niches and the capacity of well-organized genomes to accommodate new genes, while selection at the community level might contribute to the maintenance of MGEs that reassort supraspecific pools of accessory genes across organisms. Overall, these processes enable the astonishing diversity of the bacterial world.
